# Preference for hotline versus mobile application/countdown-based mobile overdose response services: a qualitative study

**DOI:** 10.1186/s12954-024-00944-9

**Published:** 2024-02-05

**Authors:** William Rioux, Adrian Teare, Nathan Rider, Stephanie Jones, S. Monty Ghosh

**Affiliations:** 1 Department of Medicine, Faculty of Medicine and Dentistry, University of Alberta, Edmonton, AB Canada; 2 College of Medicine, University of Saskatchewan, 107 Wiggins Rd, Saskatoon, SK S7N 5E5 Canada; 3 Department of Medicine, Cumming School of Medicine, University of Calgary, 3330 Hospital Dr NW, Calgary, AB T2N 4N1 Canada; 4 Three Hive Consulting, Vancouver, BC Canada; 5 Department of Internal Medicine, Faculty of Medicine and Dentistry, University of Alberta, Edmonton, AB Canada

**Keywords:** Overdose, Drug poisoning, Virtual overdose monitoring services, Harm reduction, Supervised consumption, Public health

## Abstract

**Background:**

In response to the exacerbated rates of morbidity and mortality associated with the overlapping overdose and COVID-19 epidemics, novel strategies have been developed, implemented, operationalized and scaled to reduce the harms resulting from this crisis. Since the emergence of mobile overdose response services (MORS), two strategies have aimed to help reduce the mortality associated with acute overdose including staffed hotline-based services and unstaffed timer-based services. In this article, we aim to gather the perspectives of various key interest groups on these technologies to determine which might best support service users.

**Methods:**

Forty-seven participants from various interested groups including people who use substances who have and have not used MORS, healthcare workers, family members, harm reduction employees and MORS operators participated in semi-structured interviews. Transcripts were coded and analyzed using a thematic analysis approach.

**Results:**

Four major themes emerged regarding participant perspectives on the differences between services, namely differences in connection, perceived safety, privacy and accessibility, alongside features that are recommended for MORS in the future.

**Conclusions:**

Overall, participants noted that individuals who use substances vary in their desire for connection during a substance use session offered by hotline and timer-based service modalities. Participants perceived hotline-based approaches to be more reliable and thus potentially safer than their timer-based counterparts but noted that access to technology is a limitation of both approaches.

## Highlights

1. Participant preferences varied regarding the desire for connection to peers through mobile overdose response services and as a result preferences of hotline versus timer-based services.
2. Participants believed that hotline services would be safer than their automatic timer counterparts
3. Access to technology is a major limitation of these services, and participants note that the varied access pathways promote accessibility of services.
4. The physical requirements of timer-based applications may pose additional challenges for people who use drugs by injection.
5. Participants made additional suggestions to improve both service modalities including hands-free timer refreshing, video calling methodologies and creation of online harm reduction communities.

## Introduction

Since the recognition of the overdose epidemic as a public health emergency in Canada in 2016, rates of fatal overdoses have only continued to soar. Fueled and exacerbated by various factors associated with the COVID-19 epidemic, mortality rates have doubled in the past 4 years [1]. Mandatory isolation measures, reductions in operating hours or closing of harm reduction services coupled with increasing toxicity of the drug supply contaminated with fentanyl and its more potent derivatives, benzodiazepines, xylazine and nitazene; have all contributed to the rising healthcare and life costs [2–4]. To address these concerns, people who use substances (PWUS) and public health officials have created novel strategies aimed at preventing fatal overdoses.

**Fig. 1:**
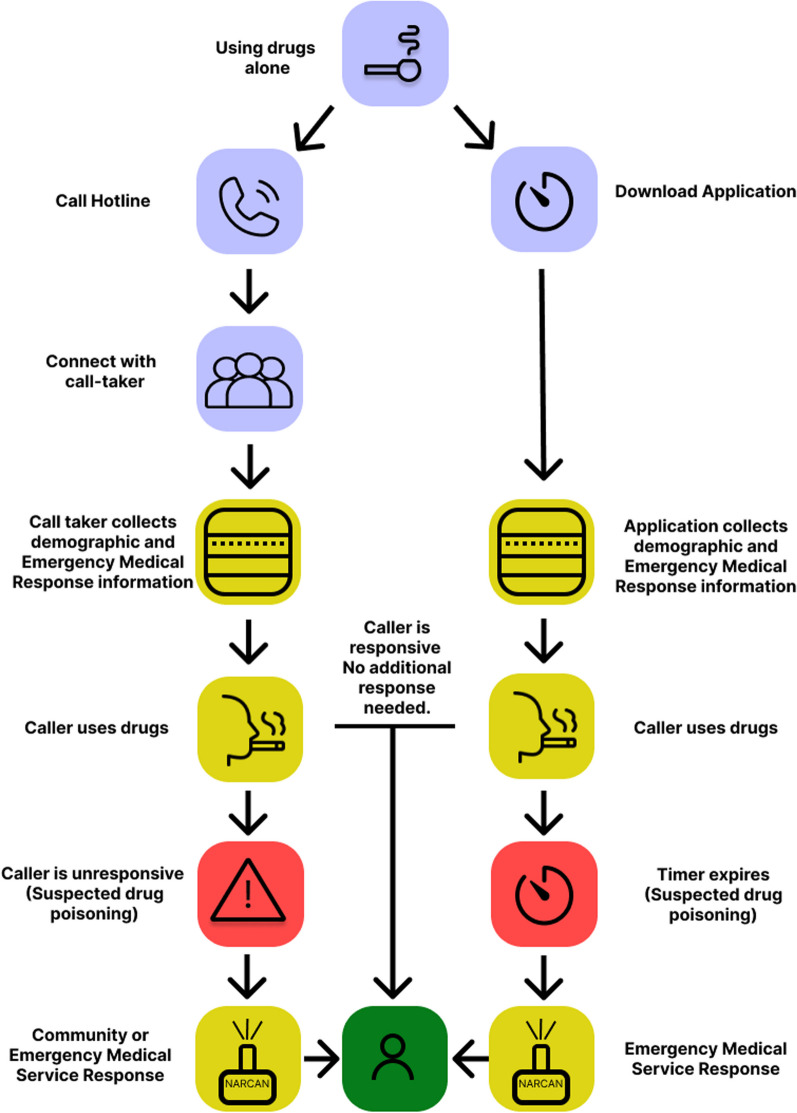
MORS description

One such strategy has been the use of virtual overdose monitoring services, more recently termed mobile overdose response services (MORS) [5]. These services are designed to help mitigate a variety of barriers facing individuals who use alone, including those who use in solitude due to lack of access to physical supervised consumption sites (SCS), stigma and unavailability of SCS' that support the individuals’ route of choice [6]. MORS enable any individual with access to a phone and connection (including via internet, landline or cellular services) to access a mobile version of SCS. Within the MORS category, two main forms of MORS exist and are compared in Table 1. These services are hotline-based services, where individuals are connected to an operator via a phone number, and smart device timer-based services, where clients can connect with operators or an automated countdown via the app, both of which are used in tandem with a drug use session and serve to enact a more rapid emergency response should an individual become unresponsive. Not included within our definition of MORS or within this study are similar technologies such as overdose buttons and reverse motion detectors [7]. See Fig. 1 for a basic explanation of service operation.

**Table 1:** Comparison of hotline and application-based services

Charecteristics	Hotline services	Timer services
Brief description of the service	Hotline-type services connect people who are using substances to live service operators. These service operators will stay on the line while someone uses their substances and will call either emergency medical services or designated community responders to intervene in the event of an overdose [8–10]	Timer-based services remain unmonitored and function in similar ways to an egg timer. An alarm begins quietly and increases in volume until a set time has passed. Once the time has passed, the application will connect with emergency medical services and have paramedics or other emergency services respond to the user's location [11, 12]
Technology requirements	Hotline services like the National Overdose Response Service (NORS) and Never Use Alone (NUA) hotline only require a phone and connection to cellular or landline services [8, 10] Other services like Brave require a connection through the Internet or utilization of smart device data [9]	Of the timer-based services currently on the market in Canada, both Lifeguard and the Digital Overdose Response Service require an Internet connection or connection through cellular data which may limit access for certain populations [11, 12]
Availability	The Brave App is available globally The National Overdose Response Service is available across Canada The Never Use Alone hotline is available in most parts of the United States	Due to the linkage of these services with existing emergency medical dispatches, these services are only available province-wide The Connect by Lifeguard app is only available in British Columbia and parts of Ontario, Canada The Digital Overdose Response Services is only available in Alberta [11, 12].
Operation	To our knowledge, all of these services are run by people with lived and living experience of substance use sometimes referred to as peers [9, 13]	Of the services currently available, all timer-based services are government or private industry-run and operated [11, 12]
Additional services offered	Due to the nature of hotline services, many callers also utilize the service for peer support, mental health service calls and connection to resources [14, 15]	No additional live services offered within the app directly, but resource links are provided for users [9, 12].
Current research	To date, only two peer-reviewed publications are available describing NORS with no deaths reported [14, 16, 17]	To date, evidence released by both DORS and Lifeguard describes usage statistics but does not report on the number of deaths averted while using the service. [18, 19]
Costs:	All services are free of charge to users and customers	Both mobile applications are free of charge to users and customers

While these services both provide different approaches to MORS, there are inherent strengths and weaknesses of each service type. Additionally, there likely exist differences around preferences for these services by various vested interest groups. Indeed, many of these services vary in their accessibility by location, requirements for internet connection and services offered. With some exceptions, hotline services are offered nationally without requirements for internet connection and are operated by people with lived and living experience and have a few peer-reviewed studies reporting outcomes [14, 16, 17]. In contrast, currently available timer-based services are offered within select provinces, are government-run and to date have no peer-reviewed publications regarding their effectiveness [17]. The aim of this study was to further understand the perspectives of various vested interest groups in the utilization, operation and delivery of MORS. These perspectives would help provide further context and insight around the pros and cons of these services, and the impact it may have on their utilization.

## Material and methods

We conducted semi-structured interviews with 47 participants from various different vested interest groups, including PWUS who were both familiar (*n* = 12) and not familiar (*n* = 8) with MORS, healthcare professionals (*n* = 10), in-person harm reduction sector employees (*n* = 6), MORS employees (n = 6) and family members of PWUS (*n* = 5). Participants were recruited through a combination of convenience and snowball sampling for groups that were identified a priori based on vested interests in the program. A core advisory group consisting of the PI who is also a healthcare professional, two MORS administrators with lived experience and a MORS operator created a list of key individuals and/or groups across Canada encompassing each representative group. These included groups that represent PWUS, MORS service providers and agencies that provide harm reduction. Once identified, SJ independently reached out to them for voluntary participation in the study. Each individual or organization recruited was then requested to suggest other individuals or groups who could be potential candidates for participation. The study received approval from the University of Calgary Conjoint Health Research Ethics Board (REB21-1655). The consolidated criteria for reporting qualitative research (COREQ) framework was used to guide the reporting of results.

### Inclusion and exclusion criteria

In conjunction with needing to be a part of one of the previously mentioned vested interest groups, participants were also required to be 18 years of age or older, reside in Canada and be able to communicate in English to qualify for interview participation. All participants were provided with descriptions of MORS available in Canada as part of the interview package and had these services explained prior to their interviews to ensure similar baseline levels of understanding. Individuals would be excluded if they were unable to provide meaningful consent; however, none of the invited participants met this exclusion criteria. No participants were excluded from the study. Interviewees with lived and living experience of substance use were provided a $50 Visa gift card as honoraria for their participation.

### Interviews

We created interview guides in collaboration with people with lived and living experiences of substance use in addition to MORS operators. Questions deemed important to understanding the differences between the MORS was first solicited by MORS operators, service users and administrators, from which detailed interview guides were constructed. Key individuals were then reengaged with the completed interview guide to ensure they captured the context of these key questions while also utilizing appropriate language. Interviews were conducted by a third-party research consulting firm which consisted of two female evaluators with master’s level training (SJ, LA) between February and March 2022, and both evaluators were compensated for their work. Each interview was approximately 20 to 60 min in length, and interviews were completed on the phone. There was no previously established relationship between evaluators and interview participants, and only the interviewer and interviewee were present on the interview call. Repeat interviews were not conducted, and interview field notes were not taken. Interviews were recorded using TapeACall and transcribed using a third-party transcription service.

### Coding

Qualitative data were encoded via thematic analysis to identify themes that could help organize the perceptions and opinions shared by study participants [20]. On the first three transcripts, coding was compared by the two evaluators to refine a codebook and ensure consistency, after which evaluators coded the transcripts independently, by utilizing the refined codebook. To ensure coding congruency, each evaluator reviewed transcripts coded by their counterpart through Dedoose. A codebook was developed and updated in real time based on joint evaluator agreement. Disagreements were discussed between each evaluator to achieve consensus. Following the initial coding, coded quotations were reviewed with the consulting project manager (KM, with advanced training in qualitative methods). Thematic saturation was sought for key themes using the framework described by Glaser and Strauss, and triangulation was conducted with the primary investigator and two peers MORS Service operators [21].

## Results

From our key interest groups, participants represented a wide variety of provinces and territories and resided in both urban (*n* = 40 (85%)) and rural (*n* = 7, (15%)) locations. Gender data were only collected for both groups of PWUS and family members, of these 13 (52%) identified as women, 11 (44%) as men and 1 (4%) as non-binary. Overall, many perceived strengths and limitations of timer and hotline-based MORS were discussed by participants. Service preferences were broken down into four main themes: (1) sense of connection with others, (2) perceptions around individuals’ privacy while using the services, (3) perceived safety while using each type of service, and (4) accessibility. Participants’ preferences were highly individualistic and therefore present unique perspectives and preferences regarding the use of each service modality. These themes are summarized in Table 2.

**Table 2:** Key themes of hotline versus timer-based services

	Hotline services	Timer services
Sense of connection	Participants note that this option provides a connection to operators for those who may feel isolated Participants note that some may be uncomfortable with talking with a different operator each time they call Participants suggested that video calls would more conducive to building trusting relationships Building a community in which MORS users could interact was also recommended	Timers be more appropriate for those who preferred to use by themselves and did not want to connect with others or wanted added privacy Building a community in which MORS users could interact was recommended
Perceptions of safety	Participants perceived hotline-based services to be a more reliable and safer option due to decreased reliance on technology and faster response times	Participants perceived timer-based applications to have additional points of technological failure particularly due to requirements for internet connection.
Perceptions around privacy	Participants who had previously used hotline-based MORS note that these services respect their privacy	People who use substances were apprehensive about using application services as most available within the region (Alberta and British Columbia) are government-affiliated Clarity on collection, use and storage of personal data would be helpful for these services
Accessibility	As highlighted by participants, technology access would undoubtedly pose a limitation in terms of access to services Cell phone plans may be required to access services, toll-free numbers are recommended to address this barrier Reductions in the automation of initial connecting messages for hotline-based services were suggested Texting was seen as a helpful feature due to some individuals have limited data plans but unlimited texts. Chat rooms features could be helpful to build connection and support	As highlighted by participants technology access would undoubtedly pose a limitation in terms of access to services Internet connection requirements may pose an additional limitation for the use of this service modality The task requirement of clicking a button may pose a challenge for those who inject their substances. Participants suggested that voice recognition may be better suited as a mechanism for refreshing the timer, particularly for those who use injection routes of administration

### Theme 1: Connection

Participants presented mixed perspectives regarding their desires for a personal connection with the various services available. While some participants preferred the personal connection offered by hotline-based services in which one spoke to an operator, others discussed how app-based services may be more appropriate for those who preferred to use by themselves and did not want to connect with others or wanted added privacy. In regard to hotline-based MORS, participants stated:

*"I have talked to one or two people about just stuff I was going through, which was great. And they were really good about that, just to listen. Yes, and they’re pretty good too, when I was nodding off and stuff, they’d keep me talking – just distract me about stupid shit too, which was great."*—Female PWUS with MORS experience.

Another took a more direct approach.

*"I can just call, do my stuff, and then hang up"*.—Female PWUS with MORS experience.

This is echoed within individuals who utilize timer-based services.

*"You can just log in and set a timer and you know, then you don’t have to talk to anybody"—Female PWUS with MORS experience.*

One respondent speculated possible gender differences regarding the appropriateness of both forms of MORS; for example, women may be more comfortable with talking to someone over the phone, while men may be more likely to use the app due to “*hav*[ing] *a harder time opening up and talking to somebody*”—Healthcare provider.

One of the limitations of hotline-based services recognized by interviewees was that clients often were not connected to their preferred operators due to the number of staff and volunteers who operate the service.

*“with (one service)as an example, you’ve got a whole host of volunteers and staff that are on different times of the day […] you might get a different person that you talk to every time […] for some people they might be OK or comfortable with that. For other people, they might do better building a relationship with one individual”*—MORS operator.

Many participants described very specific features that they preferred or desired when it came to MORS. For instance, chat features whether with an individual operator as a text/SMS aspect or as a group chat room were considered potentially helpful for individuals using substances, creating further connection and community:

"*But is there even an option for some kind of SMS-based safe consumption-based service, because I’ve heard that from patients where, oh I don’t have phone minutes and I don’t have data, but I have unlimited text. I would say SMS probably is the most accessible in terms of technology"*—Healthcare provider.

"*There was a lot of online forums that started to pop up around many different interests. And people started to create either chat rooms or connect with each other on these things in order to keep each other safe. And so, I think that community aspect could be really valuable in relationship to using something like (hotlines) or the different apps"*—Male PWUS with MORS experience.

Lastly, participants suggested that if individuals were interested in connecting with others, video chat options should be available. Respondents discussed how “*body language is such a huge part of a conversation that indicates how someone feels about something*” (Male PWUS with MORS experience), so adding a video option could help to provide a greater connection with the operator—stating, that it is “*easier to build rapport and community with someone when you can see their face*” (Male family member of PWUS). Participants did, however, acknowledge that there may be additional privacy concerns for both service users and operators should this modality be implemented.

### Theme 2: Privacy

Of those who had previously used MORS, most felt that their privacy was respected and may mitigate concerns of recognition and stigma over accessing in-person supervised consumption services. However, concerns were raised regarding the organizations which operated various services alongside the data collected.

“*I am a little hesitant about the (timer service), just because there is so much – I don*’*t know if secrecy is the right word. It just seems suspect in the way that the government wants to collect personal health information. Is it being used for surveillance? Is it being linked to other healthcare supports? We just don*’*t know; it hasn*’*t been very forthcoming with the information and data being collected.”*—Community harm reduction provider.

In reference to one of the hotline-based MORS, one participant mentioned the following.

*“If you don*’*t want to give your name, that*’*s fine, you just give a code. And you tell them where you’re located, and you tell them what you’re taking and how. And they stay on the line while you are taking it. So it is very, very easy to use.”*—Female PWUS with MORS experience.

Overall, participants highlighted that clarity on the collection, use and storage of personal information would go a long way toward allaying some of the fears of using MORS. Similarly, many individuals expressed concerns regarding police involvement and surveillance, particularly when it came to child care, enforcement of an existing warrant or confiscation of substances.

“*I think if it was now, like I have a daughter, I would be too afraid that I would lose my kid sort of thing, so that would be the scariest. Or get arrested, find out like a warrant, you know, if the paramedics show up do the police come.*”—Female PWUS with MORS experience.

Many participants suggested confidentiality agreements and non-disclosure agreements should be implemented to ensure that only appropriate information is shared with emergency medical services or the criminal justice system.

### Theme 3: Perceived Safety

In general, participants perceived hotline-based services to be more reliable and safer in the event of an overdose. Responses were perceived to be more rapid and less fraught with technical issues despite service users of both modalities reporting various dropped calls or other technical issues.

“*it was easier to be helped had anything gone wrong* […] *it’s that quicker, more immediate response*”—Female PWUS with MORS experience..

Despite their lack of service usage, PWUS who had not used MORS believed that application-based services would have more technical difficulties.

*“relying on Internet connection when using the app may pose safety challenges if the connection is dropped or unstable*”—Female PWUS without MORS experience.

*“The personal aspect of speaking to someone over the phone (e.g., not being automated) provided a level of comfort and access to additional support and resources if needed.”*—Male PWUS with MORS experience.

Overall, participants perceived the hotline-based modalities to be the safer option for their substance use session. In contrast, participants believed the GPS system associated with the applications may be helpful in locating individuals if they have an overdose event. This, of course, would need to be balanced with concerns around privacy and collectively navigated with the service users.

### Theme 4: Accessibility

Another theme that emerged from the analysis of the qualitative interviews was the accessibility of the various MORS modalities. While access to technology may be a major barrier to service access, those that require internet access through Wi-Fi or data may pose additional barriers to service users. When discussing accessibility participants offered “*toll-free number*(s)* like 911*” would allow individuals to be able to access services without internet connection, especially as it would be free of charge. “*Maybe you don’t need Wi-Fi or—I mean that’s huge. Like that would be a big hurdle*”—Male PWUS with MORS experience. Furthermore, individuals also discussed the logistical challenges of multitasking while using substances.

*“With the (timer based application), you have to refresh the timer every 30 seconds, like just click that you OK and when you’re shooting up it’s kind of a two-handed operation, you know? Because you have to have one arm out with the tourniquet and then you have to use the other hand to be inserting the needle, so it’s a little bit difficult to constantly be like clicking on the app whereas with (hotline) you don’t need to do that; you just have to talk.”— Female PWUS with MORS experience.*

One of the largest limitations to the accessibility of these services discussed by participants was undoubtedly individuals’ lack of access to technology. In regard to barriers to accessibilty, one participant stated the following: *“I think it would be the hardware access like being able to actually, you know, get on the internet or have access to a phone, things like that.”—Healthcare provider.*

Overall participants mentioned that the two types of services (hotline and automated) existing at the same time “*really helps to fill both niches of people who need those services*” (Female PWUS with MORS experience). The relative strengths and weaknesses in the accessibility of each service allow for the greatest number of users to feel comfortable accessing these virtual harm reduction services. Innovative ideas were presented to expand the acceptability of various services.

In regard to suggestions for increasing the accessibility, several respondents discussed the need to have less automated starts to the calls for hotlines.:

"*Everybody is trained and professional and they’re courteous and they’re helpful really so wouldn’t know how to make it easier; maybe just that you don’t have to wait as long through the first automated message. That’s all I could see, right; some people are in a hurry, like really"*—Female PWUS with MORS experience.

Similarly, to increase the accessibility of timer-based services, one participant suggested that the use of voice activation when using the timer might better support clients who need to use both hands when administering their substances and struggle to turn off the alarm (Female PWUS with MORS experience).

Overall, participants discussed their preferences regarding connection, safety, privacy and accessibility offered by various MORS. In addition, suggestions were made regarding potential ways in which barriers may be addressed and services may be improved.

## Discussion

While the appropriateness and effectiveness of formalized MORS have been studied across recent literature [14, 16, 17, 22, 23], this is the first study to gather perspectives on the two common types of currently available cell phone-based mobile overdose response modalities. Overall, the results from our qualitative exploratory study show differences in preferences for human connection, perceptions of privacy, beliefs about safety and ideas about accessibility.

As highlighted by interview participants, multiple service options would cater to the multitude of preferences for connection while using substances. It was further hypothesized that there may be gender-specific use preferences in regard to these services. Indeed virtual spotting (such as that provided by hotline-based services) has been previously speculated to create a safer space for women and gender minorities who face increased rates of domestic violence and stalking behaviors when accessing SCS [24]. Previous studies of one MORS service (the National Overdose Response Service ) have found similar results in that the majority of the individuals using the services identified as women and gender minorities made up a significant proportion (81.9%) of service users who reported their gender [25]. While there is a dearth of literature on the demographic-specific use of timer-based MORS, additional efforts should be conducted to promote MORS to males who continue to make up the majority (74%) of overdose deaths in Canada [1].

To our knowledge, no studies have examined the impacts of telephone-based peer support; however, in-person peer support has demonstrated significant positive effects, including creating a trusting environment, providing overdose prevention education and improving access to social services [26]. The capacity to provide these additional services in a tailored, interactive fashion instead of providing links to online resources could be a key advantage to the hotline-based services. Lastly, some potential quality improvement initiatives or features which may be considered during the establishment of services include video-based communication for hotline services and voice-based timers to reduce some of the barriers to connection and service use raised by study participants.

Regarding individual perceptions of safety, our results demonstrate that both groups of PWUS who have and have not used MORS believe that hotline-based services would be a safer option when enacting an emergency response; however, only a few participants in this study had experience using application-based services. To date, data pertaining to the safety of only one hotline-based service have been published in peer-reviewed journals [16, 25] and there is no literature available on the efficacy of timer-based applications [6, 27]. Timer-based applications have largely been government or privately operated, and no data have been made available regarding any potential rates of fatal overdoses despite various statistics on service usage being available in media releases [18, 19]. Furthermore, with the diversity in service modalities available, formal mechanisms for ensuring the quality and safety of service provision are warranted as failures can result in potentially fatal consequences for PWUS [6]. Additional research, particularly in relation to timer-based services for which there is a dearth of literature, should be conducted to demonstrate the efficacy of various MORS services in order to provide PWUS with a greater understanding of the potential reduction in mortality offered by these services.

The preservation of privacy was a key theme identified by participants. While hotline-based services such as the National Overdose Response Service and Never Use Alone do not store identifiable health information, other apps like Lifeguard, Brave and the Digital Overdose Response Service may store sensitive information such as names, dates of birth and addresses which could theoretically be compromised in the event of a cyberattack [8–12]. Indeed, the criminalization of substances is a continued theme that is seen across the literature, with many individuals expressing concerns regarding the loss of child custody, employment and the limitations of the Good Samaritan Act (as it relates to the execution of a warrant) with police attended overdoses [24, 28, 29]. Similarly, data collection and surveillance practices which are employed within harm reduction programs have been argued to potentially result in decreased service uptake and widening of health inequalities in PWUS [30, 31]. Improving data security and offering transparency regarding the purpose for data collection and the circumstances for which it may be used and disclosure might help alleviate privacy concerns. Future collaboration and integration of PWUS in decision making regarding service use outcome measures are recommended to ensure the best interests of PWUS and MORS users.

Lastly, in terms of accessibility, participants believed phone hotline-based services were slightly more accessible due to their more limited requirements in terms of stable internet access; however, both are limited by the hardware requirements for PWUS. Undoubtedly, equitability of access remains a challenge for many individuals, particularly PWUS. Quantitative research conducted across multiple SCS in the province of British Columbia noted that more than half of clients had no reliable phone access [32]. A more recent review found that between 79 and 96% of individuals with substance use disorders had phones, with greater than 60% of individuals using these to access the internet or applications [33]. Smartphone ownership and literacy, however, have also been demonstrated to be lower among low-income, racial and ethnic minorities, and basic text messaging or talk (as seen in hotlines) was recommended to reach populations in “urban safety-net outpatient settings” [34]. The same study states that applications would be applicable to “younger and relatively wealthier” individuals [34], and as a result may appeal to those who may be dissuaded from accessing in-person services for fear of stigma, loss of employment or child custody [24, 32]. While this is a broader system-based issue, a focus on improving access to these technologies and improving literacy around technology use would be key to ensuring improved uptake of these services and improving overall access and utilization equity. In contrast, technology has been identified as having the potential to play a considerable role in reducing health disparities for those living in rural communities in individuals receiving mental health and substance use treatment [35]. Indeed, harm reduction services are often limited to larger urban settings and have a limited radius of effectiveness (500 m from the site) [6, 36–38]. Lastly, it should also be noted that currently, due to the nature of the timer-based services, these services are province-specific (with the Digital Overdose Response Service and Lifeguard being available in within three Canadian provinces: with the former available in Alberta and the latter available in British Columbia and Northwestern Ontario further limiting their reach [11, 12]. Evidently, the most glaring challenge to the accessibility of MORS in general is access to a phone with a calling plan, data or Wi-Fi; however, these services may increase access to harm reduction for a large proportion of PWUS who may not have previously had the opportunity to access in-person services. Due to the previously studied economic benefits of harm reduction programs including supervised consumption sites [39–41] and hotline MORS [22], potential future modeling and cohort studies should examine the health, economic and social impacts of phone provision for PWUS.

Overall, hotline-based services were perceived to provide a greater sense of connection for those who found it valuable. Additionally, participants perceived additional service reliability offered by hotline services may increase safety for PWUS using MORS. This study highlighted that the current diversity of service modalities available to users appeals to multiple types of users.

### Limitations

Interpreting the results of our study requires consideration of a few limitations. The convenience/snowball nature of the sample may have limited the diversity of opinions, despite efforts to recruit participants from diverse geographic and demographic backgrounds. It is possible that the sample does not reflect the opinions of some of the individuals targeted by MORS (e.g., employed individuals who use substances alone at home) and results may not be generalizable outside of Canada. Lastly, the results do not prove the effectiveness of the two main types of MORS to PWUS but only demonstrate stakeholder perceptions of appropriateness and certain best distribution practices. They also cannot compare the effectiveness between the two modalities, and additional transparency on behalf of MORS organizations in regard to data surrounding the efficacy of these services is required to ensure that individuals are informed as to the risks and benefits of using these services versus using alone. In conjunction, future studies should be conducted to help inform people who use substances and others in their community regarding their use of these services.

## Conclusions

The results of this study can be used to inform public health decision-makers, local advocacy groups, addiction professionals and governments of all levels regarding the MORS they would like to have within their own jurisdictions. Our findings illustrate the various strengths and limitations of staffed hotline-based services and unstaffed application-based services on PWUS. Considering these various perspectives when developing novel harm reduction strategies can help to optimize service delivery and subsequently the mortality associated with the contamination of illicit substances.

## Data Availability

The data that support the findings of this study are available on request from the corresponding author, M.G. The data are not publicly available due to the sensitivity of substance use and interview transcripts containing information that could compromise the privacy of research participants.

## Abbreviations

MORS: Mobile overdose response services
PWUS: People who use substances
NORS: National Overdose Response Service
DORS: Digital Overdose Response Service
NUA: Never Use Alone
